# *Drosophila* Cuticular Hydrocarbons Revisited: Mating Status Alters Cuticular Profiles

**DOI:** 10.1371/journal.pone.0009607

**Published:** 2010-03-09

**Authors:** Claude Everaerts, Jean-Pierre Farine, Matthew Cobb, Jean-François Ferveur

**Affiliations:** 1 Centre des Sciences du Goût et de l'Alimentation, UMR-6265 CNRS, UMR-1324 INRA, Université de Bourgogne, Agrosup, Dijon, France; 2 Faculty of Life Sciences, University of Manchester, Manchester, United Kingdom; INRA - Paris 6 - AgroParisTech, France

## Abstract

Most living organisms use pheromones for inter-individual communication. In *Drosophila melanogaster* flies, several pheromones perceived either by contact/at a short distance (cuticular hydrocarbons, CHs), or at a longer distance (*cis-*vaccenyl acetate, cVA), affect courtship and mating behaviours. However, it has not previously been possible to precisely identify all potential pheromonal compounds and simultaneously monitor their variation on a time scale. To overcome this limitation, we combined Solid Phase Micro-Extraction with gas-chromatography coupled with mass-spectrometry. This allowed us (*i*) to identify 59 cuticular compounds, including 17 new CHs; (*ii*) to precisely quantify the amount of each compound that could be detected by another fly, and (*iii*) to measure the variation of these substances as a function of aging and mating. Sex-specific variation appeared with age, while mating affected cuticular compounds in both sexes with three possible patterns: variation was (*i*) reciprocal in the two sexes, suggesting a passive mechanical transfer during mating, (*ii*) parallel in both sexes, such as for cVA which strikingly appeared during mating, or (*iii*) unilateral, presumably as a result of sexual interaction. We provide a complete reassessment of all *Drosophila* CHs and suggest that the chemical conversation between male and female flies is far more complex than is generally accepted. We conclude that focusing on individual compounds will not provide a satisfactory understanding of the evolution and function of chemical communication in Drosophila.

## Introduction

Pheromones are chemical signals that mediate inter-individual communication in most animals and plants. In vertebrates and invertebrates, many molecules—perceived by olfactory and gustatory systems—influence various behaviours including courtship and mating [1]. In *Drosophila melanogaster*, as in many dipterans, most known sex pheromones are cuticular hydrocarbons (CHs) [2]. CHs probably initially served as a protection against environmental factors (desiccation [3], [4] or entomopathogens [5]). Some of these compounds now function as species-specific signals (pheromones), providing both inter- and intraspecific information [6]. In *D. melanogaster*, long-chain hydrocarbons on the adult fly cuticle are perceived by contact or at a short distance by other flies [7], [8]. Despite over a quarter century of intensive investigation [9], our understanding of the role of these substances in *Drosophila* chemical communication remains rudimentary. Some of these cuticular hydrocarbons (CHs) show a marked sexual dimorphism: only female flies produce CHs with two double-bonds (often 7,11-dienes) which stimulate male courtship, while monoenes (with one double bond, such as 7-tricosene; 7-T) are mostly found on males [7], [9], [10]. These monoenes tend to inhibit male courtship [8], [9], [11] and increase female receptivity [12]. Minor CHs also play important pheromonal roles: 5-tricosene (5-T) is thought to inhibit male courtship while 9-pentacosene (9-P) enhances copulatory behaviour [13], [14].

Evidence from our laboratory suggests that known CHs explain only one third of male courtship, with volatile substances playing an equal role, and unknown stimuli accounting for a final third [15]. Little progress has been made in identifying these other factors - the only volatile compound thus far identified as important in courtship behaviour is *cis-*vaccenyl acetate (cVA), which was initially described 40 years ago [16]. This non-CH molecule, which has recently been the subject of intense investigation [17], [18], is only one component in Drosophila chemical communication, and is transmitted by the male to the female during ejaculation; it strongly inhibits male courtship [19], [20] and stimulates female mating [17]. Recently, a new oxygenated compound that inhibits male courtship, CH503 (3-O-acetyl-1,3-dihydroxyoctacosa-11,19-diene), has been found in the male ejaculatory bulb and has been shown to be transferred to female during mating [21]. However, there is no consistent evidence that either cVA or CH503 has any behavioural role prior to being released during mating.

The other stimuli involved in the control of Drosophila courtship and mating are unknown. In fact, despite the amount of work on the subject, we have a very partial view of the CHs present on the Drosophila cuticle. In general, only one analytical technique has been used - solvent extraction followed by gas chromatography (GC) sometimes coupled with mass spectrometry (MS) [9], [22], [23], although recently both DART-TOF-MS [24] and UV-LDI-o-TOF MS [21] have been employed. All three approaches provide a partial and non-congruent description of the fly's cuticular profile and how it changes with time and experience. The classic GC-MS technique provides quantitative estimates of the levels of each compound but kills the individual fly; DART-TOF-MS leaves the fly intact but does not describe the position of unsaturated bonds, while although UV-LDI-o-TOF MS has revealed several new oxygenated compounds which cannot be detected by GC-MS, it is relatively ineffective at detecting biologically significant monoenes and alkanes, does not reveal unsaturated bonds and it kills the fly. To determine whether Drosophila harbours novel CHs and to quantify the levels of all CHs, we combined non-lethal Solid Phase Micro-Extraction (SPME) with GC-MS. SPME is a simple, solvent-free, and reliable micro-extraction technique which was initially designed for the analysis of organic compounds in the air or in the water [25], but has been used in bio-analysis (in vitro and in vivo) [26], [27]. Although SPME has been already used as an alternative to solvent extraction of CHs in insects (e.g. ants [28], [29], [30], [31], [32], wasps [33], [34], [35], termites [36]; cockroaches [37], [38], beetles [39], [40]), it has not previously been used in Drosophila. Reportedly SPME yields samples that qualitatively and quantitatively similar to those obtained by solvent extraction [29], [32], [33], [36].

Using this procedure, we tracked the quantitative and qualitative evolution of CHs on individual flies as a function of age and mating experience. We were particularly concerned to establish whether cVA was detectable on the cuticle of virgin males and could therefore act as a pheromone prior to mating. As well as providing a far richer description of the Drosophila cuticular hydrocarbon profile, we were able to identify novel putative pheromones in this model species.

## Results

### Reassessing *Drosophila* Cuticular Hydrocarbons

**Figure 1:**
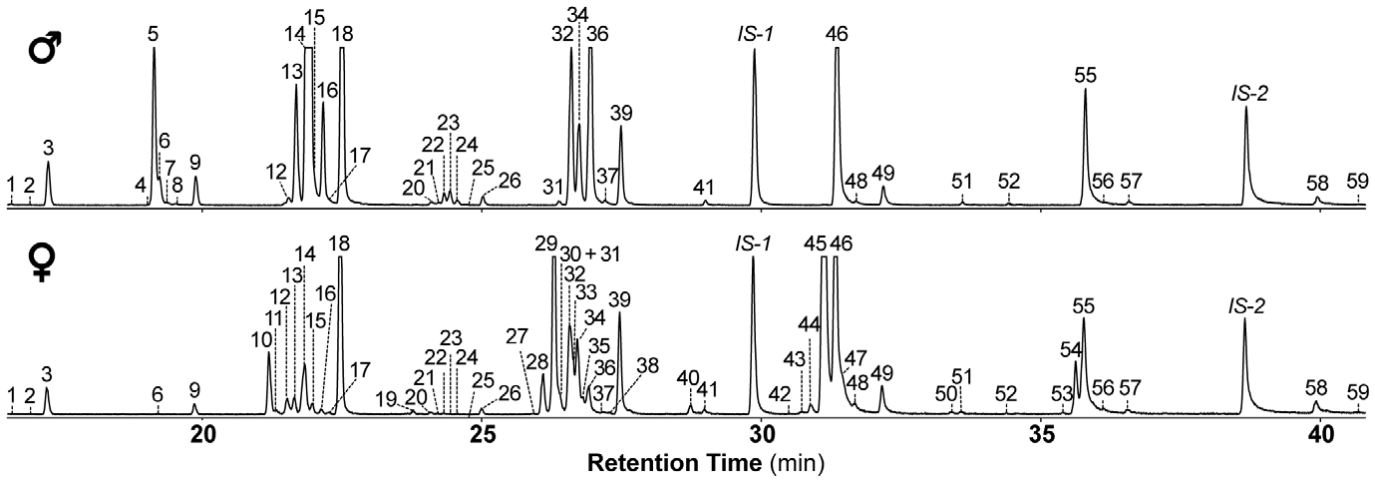
Reassessment of cuticular compounds on *D. melanogaster* flies. GC-MS chromatogram traces of a single virgin 4-day-old control male and female after whole-body extraction in hexane. The numbers above the peaks refer to the compounds listed in Table 1. *IS-1* and *IS-2* were internal standards used to calculate the absolute amounts of each compound in control males (2000±207 ng; *n* = 6) and females (2347±235 ng; *n* = 6).

We measured the cuticular profile of mature virgin male and female flies that had been isolated prior to pupation, using classic GC-MS on individual whole-fly extracts (Fig. 1). We detected 59 compounds –58 CHs (20–31C) and cVA, each of which was characterized by MS (Table 1). 19 substances were female-specific, 4 (including cVA) were male-specific and 36 were found in both sexes.

**Table 1:** Complete list of the compounds detected in whole-body extracts of 4 day old control virgin flies.

#		Abbrev.	M	F	Compound Name	KI	#		Abbrev.	M	F	Compound Name	KI
1	→	**7-He**	+	*tr*	(Z)-7-Heneicosene	2075	31	→	**12-P**	+	*tr*	(x)-12-Pentacosene	2455
2	→	**5-He**	+	*tr*	(Z)-5-Heneicosene	2086	32		**25-Br**	+	+	2-Methyltetracosane	2464
3		***n-C21***	+	+	*n-Heneicosane*	2100	33		**5,9-PD**		+	(Z,Z)-5,9-Pentacosadiene	2466
4		**9-D**	+		(Z)-9-Docosene	2169	34		**9-P**	+	+	(Z)-9-Pentacosene	2469
5		**cVa**	+		(Z)-11-Vaccenyl acetate	2172	35	→	**8-P**		+	(x)-8-Pentacosene	2474
6		**7-D**	+	*tr*	(Z)-7-Docosene	2175	36		**7-P**	+	+	(Z)-7-Pentacosene	2478
7	→	**6-D**	+		(+)-6-Docosene	2180	37		**5-P**	+	+	(Z)-5-Pentacosene	2488
8	→	**5-D**	+		(Z)-5-Docosene	2186	38	→	**4-P**		+	(x)-4-Pentacosene	2492
9		***n-C22***	+	+	*n-Docosane*	2200	39		***n-C25***	+	+	*n-Pentacosane*	2500
10		**7,11-TD**		+	(Z,Z)-7,11-Tricosadiene	2250	40		**7,11-He+D**		+	(Z,Z)-7,11-Hexacosadiene	2552
11	→	**x,x-TD**		+	Tricosadiene *	2256	41		**26-Br**	+	+	2-Methylpentacosane	2564
12		**23-Br**	+	+	2-Methyldocosane	2264	42	→	**Br-M 2**		+	branched C27 monoene	2624
13		**9-T**	+	+	(Z)-9-Tricosene	2269	43	→	**Br-M 3**		+	branched C27 monoene	2636
14		**7-T**	+	+	(Z)-7-Tricosene	2276	44		**9,13-HD**		+	(Z,Z)-9,13-Heptacosadiene	2644
15	→	**6-T**	*tr*	+	(+)-6-Tricosene	2281	45		**7,11-HD**		+	(Z,Z)-7,11-Heptacosadiene	2656
16		**5-T**	+	+	(Z)-5-Tricosene	2286	46		**27-Br**	+	+	2-Methylhexacosane	2664
17	→	**4-T**	*tr*	+	(+)-4-Tricosene	2291	47		**9-H**		+	(Z)-9-Heptacosene	2669
18		***n-C23***	+	+	*n-Tricosane*	2300	48		**7-H**	+	+	(Z)-7-Heptacosene	2678
19		**7,11-TeD**		+	(Z,Z)-7,11-Tetracosadiene	2350	49		***n-C27***	+	+	*n-Heptacosane*	2700
20	→	**24-Br**	+	+	2-Methyltricosane	2364	50		**7,11-OD**		+	(Z,Z)-7,11-Octacosadiene	2754
21		**9-Te**	+	+	(Z)-9-Tetracosene	2369	51		**28-Br**	+	+	2-Methylheptacosane	2764
22	→	**8-Te**	+	+	(+)-8-Tetracosene	2372	52		***n-C28***	+	+	n-Octacosane	2800
23		**7-Te**	+	+	(Z)-7-Tetracosene	2376	53		**9,13-ND**		+	(Z,Z)-9,13-Nonacosadiene	2846
24	→	**6-Te**	+	+	(+)-6-Tetracosene	2381	54		**7,11-ND**		+	(Z,Z)-7,11-Nonacosadiene	2855
25		**5-Te**	+	+	(Z)-5-Tetracosene	2386	55		**29-Br**	+	+	2-Methyloctacosane	2864
26		***n-C24***	+	+	*n-Tetracosane*	2400	56		**7-N**	*tr*	*tr*	(Z)-7-Nonacosene	2880
27	→	**Br-M 1**		+	branched C25 monoene	2436	57		***n-C29***	+	+	*n-Nonacosane*	2900
28		**9,13-PD**		+	(Z,Z)-9,13-Pentacosadiene	2443	58		**31-Br**	+	+	2-Methyltriacontane	3063
29		**7,11-PD**		+	(Z,Z)-7,11-Pentacosadiene	2450	59		***n-C31***	+	+	*n-Hentriacontane*	3100
30	→	**x,x-PD**		+	Pentacosadiene *	2454							

Peak numbers (#) correspond to the elution order of each compound. Abbrev.  =  abbreviated names. KI  =  Kovats index. In male and female flies, compounds were detected in quantifiable amounts (+) or in trace amounts (tr).

→  =  newly described compounds.

* =  the location of the double bonds was not determined; *x*  =  the configuration was not determined.

### Experimental Procedure Validation

**Figure 2:**
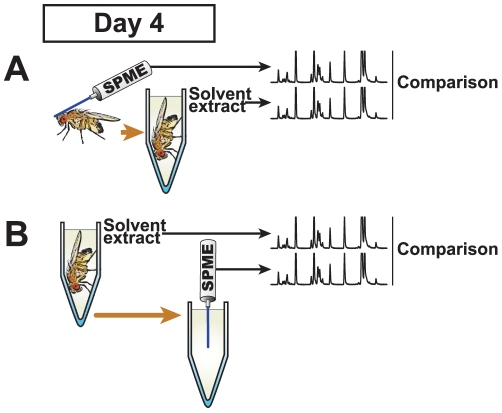
Validation of experimental procedures. The robustness of SPME was evaluated with 4 day old virgin control flies. A: Cuticular compounds sampled with SPME on individual flies which were subsequently immersed in solvent. The cuticular profiles obtained by the two methods were compared. *n* = 6–10. B: The fly was washed in solvent and the SPME fibre was immersed in the extract. The profiles produced by the two methods were compared. *n* = 6–10.

**Figure 3:**
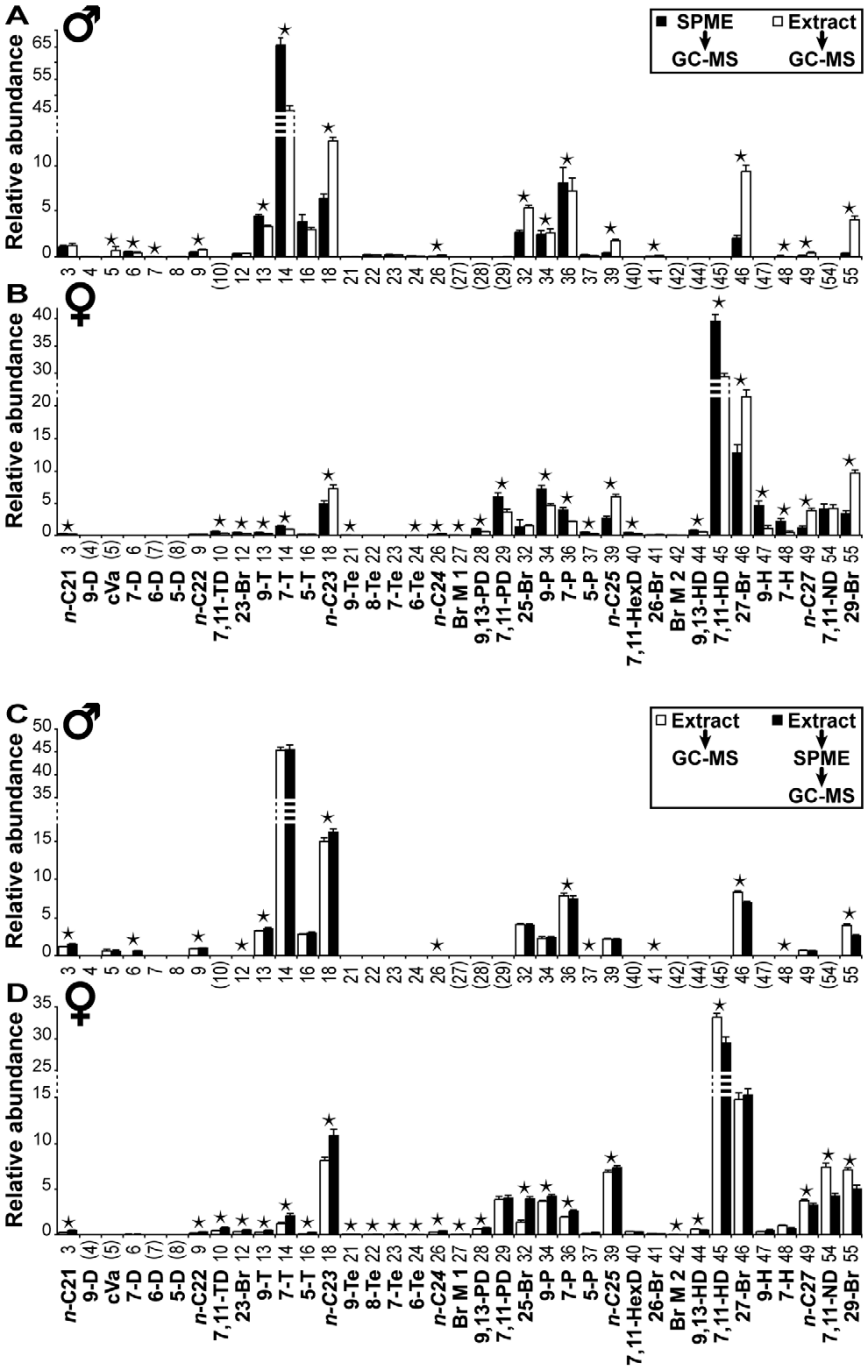
Comparison of SPME and hexane extract sampling methods in males and females. The relative abundance of compounds sampled by SPME (filled bars) or by whole-body solvent extraction (empty bars) in 4 day old virgin male (A) and female (B) flies are represented by their mean (± SEM). Only the 37 chemicals that significantly varied either with age or mating are shown. ★  =  compounds that significantly differed between the two sampling methods (p<0.05, Wilcoxon signed-rank test). The numbers and abbreviations shown below the base line refer to the compounds listed in Table 1. The numbers between parentheses were not detected in either sex (n = 8). The relative abundance of compounds sampled by direct SPME and by SPME of whole-body solvent extract. Data are shown as the mean (± SEM) of the relative abundance of compounds detected either directly in the whole-body solvent extract (empty bars) of 4-day-old virgin males (C) and females (D), or indirectly sampled by the SPME fibre immersed in the same extract (filled bars) (n = 10). Note that the cut-off limit for increasing and decreasing compounds slightly differed between males (C24/C25) and females (C25/C26). This may have been caused by the sexual dimorphism for the ratio of lighter∶heavier compounds.

To measure the effectiveness of SPME as compared to classic solvent extraction, the SPME fibre was gently rubbed on the head, thorax, wings, abdomen and genitalia of the fly; the fibre was then inserted into the GC-MS device while the fly was immediately plunged into solvent and its whole-body composition revealed by GC-MS (Fig. 2A). With the exception of cVA and CHs >29C (neither of which were detected with SPME) there were no qualitative differences–all compounds detected in one procedure were also found in the other. However, the two methods did reveal quantitative differences (Fig. 3A, B, Table 2 & 3): compared to solvent extraction, SPME generally detected higher levels of unsaturated CHs (apart from 9-P in males) and lower levels of linear and methyl-branched alkanes (except 23-Br in females).

**Table 2:** Effect of fibre polarity on the male compounds collected by SPME.

#	Abbrev.	Sample 1	Sample 2	Sample 3	Sample 4	K	*P*
**3**	***n-C21***	1.08±0.12	1.17±0.16	1.27±0.31	0.94±0.31	ns	
**4**	**9-D**	0.03±0.01	-	0.05±0.02	0.02±0.01	11.124	0.011
**6**	**7-D**	0.48±0.06	0.49±0.10	0.61±0.09	0.48±0.14	ns	
**7**	**6-D**	0.03±0.01	0.03±0.01	0.06±0.01	0.04±0.01	ns	
**8**	**5-D**	0.02±0.01	0.03±0.01	0.06±0.01	0.03±0.01	8.049	0.045
**9**	***n-C22***	0.52±0.06	0.52±0.06	0.50±0.07	0.41±0.11	ns	
**12**	**23-Br**	0.20±0.06	0.19±0.04	0.23±0.07	0.20±0.09	ns	
**13**	**9-T**	4.30±0.63	4.19±0.50	4.29±0.37	3.60±0.88	ns	
**14**	**7-T**	59.68±2.83	62.29±2.76	63.08±1.91	66.19±7.56	ns	
**16**	**5-T**	3.70±0.31	3.67±0.49	3.89±0.19	3.22±0.77	ns	
**18**	***n-C23***	7.35±0.44	7.72±0.54	7.87±0.42	6.20±1.34	ns	
**20**	**24-Br**	0.09±0.02	0.08±0.02	0.08±0.02	0.07±0.03	ns	
**21**	**9-Te**	0.07±0.02	0.05±0.01	0.05±0.02	0.06±0.02	ns	
**22**	**8-Te**	0.41±0.05	0.37±0.05	0.30±0.04	0.31±0.09	ns	
**23**	**7-Te**	0.53±0.01	0.50±0.02	0.41±0.05	0.40±0.10	ns	
**24**	**6-Te**	0.16±0.02	0.15±0.03	0.10±0.01	0.32±0.24	ns	
**25**	**5-Te**	0.03±0.01	0.04±0.01	0.04±0.01	0.02±0.01	ns	
**26**	***n-C24***	0.10±0.03	0.07±0.01	0.09±0.01	0.06±0.02	ns	
**31**	**12-P**	0.29±0.15	0.16±0.03	0.11±0.03	0.11±0.03	ns	
**32**	**25-Br**	2.66±0.33	2.23±0.24	2.50±0.42	2.56±0.73	ns	
**34**	**9-P**	2.75±0.20	2.42±0.27	2.58±0.43	2.53±0.72	ns	
**36**	**7-P**	12.17±2.62	11.01±2.71	8.75±1.50	8.09±2.32	ns	
**37**	**5-P**	0.18±0.11	0.17±0.07	0.09±0.03	0.08±0.04	ns	
**39**	***n-C25***	0.46±0.12	0.44±0.07	0.58±0.11	0.61±0.21	ns	
**41**	**26-Br**	0.03±0.00	0.03±0.00	0.07±0.01	0.05±0.02	ns	
**46**	**27-Br**	2.12±0.21	1.37±0.11	1.86±0.29	2.44±0.68	ns	
**48**	**7-H**	0.07±0.04	0.05±0.02	0.04±0.01	0.04±0.02	ns	
**49**	***n-C27***	0.08±0.02	0.06±0.02	0.10±0.02	0.12±0.05	ns	
**55**	**29-Br**	0.41±0.07	0.49±0.30	0.35±0.08	0.82±0.18	ns	

We compared the effect of fibre polarity on the male and female compounds collected by SPME, using an apolar carbowax/divinylbenzene StableFlex fibre (CW/DVB, 70 µm, Supelco, St Quentin-Fallavier, France) and a polar polydimethylsiloxane fibre (PDMS, 100 µm, Supelco, St Quentin-Fallavier, France). Both fibres were consecutively rubbed on the principal external parts of the same individual fly (head, thorax, wings, abdomen, genitalia). To avoid any effect of the first rubbing on the second SPME sampling, we swapped both sampling procedures as follows: *Sampl. 1 & 2*: first CW/DVB sampling on intact flies (*Sampl. 1*) followed by PDMS sampling (*Sampl. 2*); *Sampl. 3 & 4*: first PDMS sampling on intact flies (*Sampl. 3*) followed by CW/DVB sampling (*Sampl. 4*).

The SPME fibre was introduced into the GC-MS injection port as described in EXPERIMENTAL PROCEDURES.

Results are given as the mean (and SEM) of the relative amount of each compound (expressed in %). For each compound, the data obtained by the four sampling methods were compared using a Kruskal-Wallis test followed by Dunn's multiple pairwise comparisons (two-tailed with Bonferroni correction). Significant Kruskal-Wallis tests are shown by the K and *p* values, while the results of the subsequent Dunn's multiple pairwise comparison ares shown by the lowercase letters besides the relative amounts. The peak numbers and abbreviations refer to the compounds listed in Table 1.

**Table 3:** Effect of fibre polarity on the female compounds collected by SPME.

#	Abbrev.	Sample 1	Sample 2	Sample 3	Sample 4	K	*p*
**3**	***n-C21***	0.41±0.11	0.63±0.09	0.56±0.12	0.36±0.06	ns	
**9**	***n-C22***	0.23±0.04	0.29±0.07	0.25±0.06	0.16±0.03	ns	
**10**	**7,11-TD**	0.60±0.11	0.79±0.23	1.02±0.11	0.71±0.09	ns	
**11**	**x,x-TD**	0.07±0.03	0.10±0.02	0.11±0.03	0.07±0.01	ns	
**12**	**23-Br**	0.62±0.06	0.90±0.10	0.79±0.06	0.56±0.08	11.034	0.012
**13**	**9-T**	0.27±0.08	0.77±0.36	0.83±0.13	0.49±0.15	ns	
**14**	**7-T**	3.59±2.28	4.20±2.53	3.17±0.46	2.41±0.46	ns	
**15**	**6-T**	0.36±0.05	0.59±0.04	0.64±0.07	0.45±0.07	9.709	0.021
**16**	**5-T**	0.28±0.19	0.31±0.22	0.33±0.05	0.27±0.09	ns	
**17**	**4-T**	0.27±0.24	0.07±0.04	0.12±0.03	0.08±0.04	ns	
**18**	***n-C23***	5.02±0.79	6.13±0.82	6.38±0.98	4.91±0.55	ns	
**19**	**7,11-TeD**	0.01±0.01	0.03±0.02	0.03±0.02	0.04±0.01	ns	
**20**	**24-Br**	0.01±0.01	0.04±0.02	0.02±0.02	0.04±0.01	ns	
**21**	**9-Te**	0.01±0.01	0.03±0.02	0.01±0.01	0.02±0.01	ns	
**22**	**8-Te**	0.00±0.00	0.05±0.04	0.01±0.01	0.01±0.00	ns	
**23**	**7-Te**	0.00±0.00	0.05±0.03	-	0.01±0.01	ns	
**24**	**6-Te**	0.00±0.00	0.03±0.02	-	0.00±0.00	ns	
**25**	**5-Te**	0.00±0.00	0.00±0.00	-	0.00±0.00	ns	
**26**	***n-C24***	0.09±0.03	0.15±0.04	0.15±0.05	0.10±0.02	ns	
**27**	**Br-M1**	0.02±0.02	0.01±0.01	-	-	ns	
**28**	**9,13-PD**	0.47±0.12	1.37±0.78	0.92±0.27	0.80±0.19	ns	
**29**	**7,11-PD**	3.73±0.51	4.55±0.58	6.08±0.80	5.24±0.69	ns	
**32**	**25-Br**	1.77±0.21	1.89±0.18	2.44±0.32	2.16±0.28	ns	
**33**	**5,9-PD**	1.34±0.27	1.66±0.19	2.23±0.46	1.58±0.50	ns	
**34**	**9-P**	3.65±0.72	4.31±0.70	6.14±1.34	5.22±1.03	ns	
**35**	**8-P**	0.63±0.14	2.57±1.55	0.75±0.13	0.57±0.06	ns	
**36**	**7-P**	3.10±1.58	1.23±0.41	3.39±0.58	2.87±0.48	10.360	0.016
**37**	**5-P**	0.20±0.05	0.58±0.36	0.22±0.05	0.24±0.11	ns	
**38**	**4-P**	0.00±0.00	0.01±0.01	-	-	ns	
**39**	***n-C25***	1.82±0.23	1.91±0.39	1.89±0.21	1.77±0.33	ns	
**40**	**7,11-HexD**	0.64±0.25	0.67±0.25	0.49±0.03	0.47±0.04	ns	
**41**	**26-Br**	0.14±0.02	0.17±0.04	0.11±0.02	0.13±0.05	ns	
**43**	**Br-M3**	0.13±0.03	0.14±0.07	0.13±0.04	0.09±0.01	ns	
**44**	**9,13-HD**	0.63±0.21	0.61±0.21	0.53±0.14	0.55±0.14	ns	
**45**	**7,11-HD**	42.67±4.96	40.37±4.80	40.63±3.19	42.69±3.61	ns	
**46**	**27-Br**	8.77±1.45	7.57±1.22	7.29±0.99	7.81±1.42	ns	
**47**	**9-H**	4.91±0.44	4.51±0.43	3.89±0.34	3.96±0.32	ns	
**48**	**7-H**	1.45±0.35	1.32±0.16	1.39±0.22	1.48±0.21	ns	
**49**	***n-C27***	0.84±0.16	0.71±0.18	0.56±0.10	0.73±0.21	ns	
**50**	**7,11-OD**	0.39±0.09	1.15±0.81	0.18±0.03	0.35±0.07	8.280	0.041
**51**	**28-Br**	0.09±0.02	0.04±0.01	0.04±0.02	0.08±0.05	ns	
**53**	**9,13-ND**	0.15±0.02	0.10±0.01	0.13±0.07	0.16±0.07	ns	
**54**	**7,11-ND**	8.22±1.41	6.12±1.15	4.44±0.92	7.44±1.36	ns	
**55**	**29-Br**	2.40±0.60	1.28±0.49	1.72±0.74	2.90±1.10	ns	

*Cf*. Table 2.

To further evaluate the robustness of SPME, we used GC-MS to compare the composition of the same whole-fly extract either after a direct injection or via indirect SPME sampling, by immersing the fibre in the extract (Fig. 2B). A comparison of these profiles (Fig. 3C, D) revealed that SPME tended to reveal higher levels of the lighter compounds and lower levels of heavier compounds, but showed no difference in the identification of saturated compounds. Both methods detected cVA in males, but not in females. Furthermore, both direct injection and injection via SPME sampling allowed us to revealed >29C CHs in both sexes.

### Cuticular Profiles Change with Age

**Figure 4:**
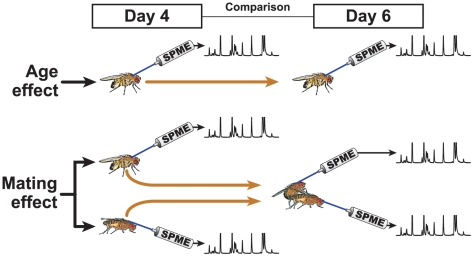
Experimental procedures to estimate aging and mating effects on CHs. To estimate the effect of aging and mating, we measaured the variation in individual flies between 4 and 6 days old. Each fly (either virgin  =  top, or mated when 6 days old  =  bottom) was sampled twice with SPME fibre. The 4 and 6 day old profiles were then compared (*n* = 6–10).

To explore the potential function(s) of the 57 Drosophila CHs, we measured changes in the profile of individual male and female flies by carrying out SPME on virgin 4-day-old flies, and on the same flies at 6 days old (Fig. 4).

**Figure 5:**
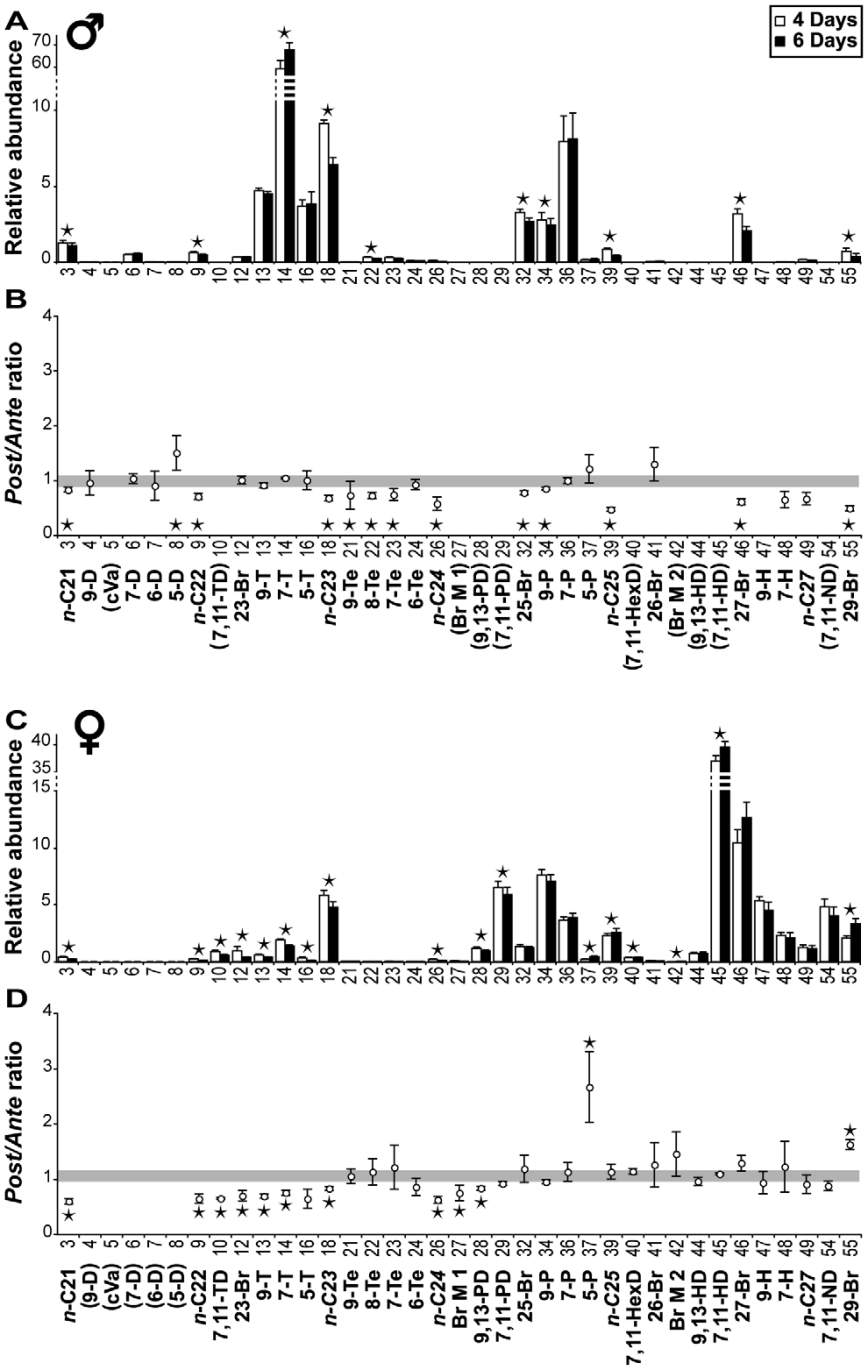
Age effects on cuticular compounds. (A & C) Global effects. Data shown represent the mean (± SEM) for the relative abundance of cuticular compounds in 4 day old (empty bars) and 6 day old (filled bars) virgin males (A) and females (C). We show only the 37 compounds that significantly varied with age or mating. The numbers and abbreviations shown below the base line refer to the compounds listed in Table 1. ★  =  compounds that significantly differed between 4 and 6 day old males (*p*<0.05, Wilcoxon signed-rank test). *n* = 6 & 8. (B & D) Individual effects. Data shown represent the mean (± SEM) for the *Post∶Ante* ratio (6 day old/4 day old) calculated for each compound in individual males (B) and females (D). The confidence limit of the ratio is shown by the shaded stripe (ranging from 0.894 to 1.078 in males, and from 0.978 to 1.168 in females). ★  =  compounds for which more than 80% individuals showed *Post∶Ante* ratios outside of the confidence limits. The compounds in parentheses were not detected in either sex.

To control for aging effects, we measured age-related changes in control flies that remained virgin (Fig. 4, upper panel). Changes in individual SPME profiles (as measured by a post/ante ratio) were considered to be significant when they exceeded the random variation observed in 80% of individuals. Small but significant sex differences were observed. In males, the amounts of most saturated and methyl-branched CHs decreased, between 4 and 6 days (Fig. 5A, B); in females, most short-chain CHs decreased while both 5-P and 29-Br increased with age (Fig. 5C, D). Three short-chain compounds, 9-Te, 8-Te, and 7-Te, did not change in 6 day-old females whereas they significantly decreased in same-age males.

### Cuticular Profiles Are Altered by Mating

**Figure 6:**
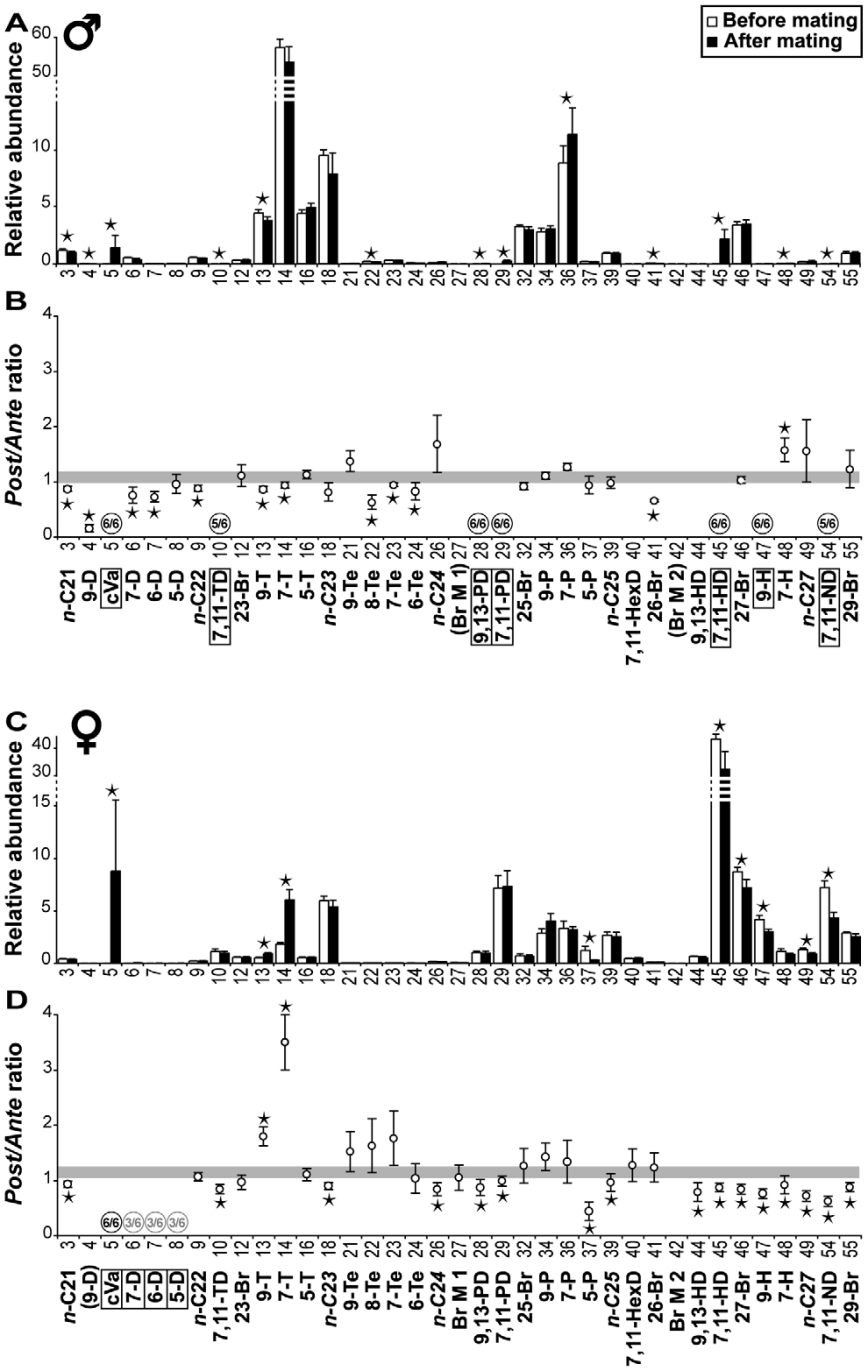
Mating effects on cuticular compounds. (A & C) Global effects. Data shown represent the mean (± SEM) for the relative abundance of cuticular compounds in 4-day-old virgin (empty bars) and in 6-day-old mated (filled bars) males (A) and females (C). *n* = 6. (B & D) Individual effects. Data shown represent the mean (± SEM) for the *Post/Ante* ratio (after/before mating) calculated for each compound in individual males (B) and females (D). The confidence limits of the *Post∶Ante* ratios calculated for the mating effect (shaded stripe) ranges from 0.968 to 1.212 in males, and from 1.037 to 1.253 in females. The compounds in parentheses were not detected in either sex; those shown within a frame appeared during mating. The numbers inside the circle (above the baseline) indicate the proportion of individuals in which they appeared; the grey circles labelled with “3/6” indicate the compounds that appeared in only 50% of mating females. For statistics, see fig. 2.

To evaluate the effect of mating, we measured the changes in the profile of flies by carrying out SPME on virgin 4-day-old flies, and on the same flies at 6 days old, following mating (Fig. 4 lower panel). Mating produced dramatic changes in CH profile. In males, mating tended to decrease the levels of 9-D, 7-D, 6-D, 9-T, 7-T, 6-Te and 26-Br, to increase 7-H, and to induce the appearance of cVA, 9-H, 7,11-TD, 7,11-PD, 7,11-HD, 7,11-ND, 9,13-PD (Fig. 6A, B–the effects of *n-*C21, *n-*C22, 8-Te, and 7-Te were excluded because similar effects were observed in virgin males). In females mating led to decreased levels of 5-P, 7-H, 9-H, 7,11-HD, 7,11-PD, 7,11-ND, 9,13-HD, *n-*C25, *n-*C27, 27-Br and 29-Br, and increased levels or led to the appearance of cVA, 7-D, 6-D, 5-D, 9-T, 7-T (Fig. 6C, D–7,11-TD, 9,13-PD, *n-*C21, *n-*C23, *n-*C24 were excluded as virgin females showed similar effects).

## Discussion

### *Drosophila* Cuticular Profiles Revisited

Among the 59 compounds that we detected in the cuticular profile of mature flies, 17 CHs were novel and have not been previously described in *D. melanogaster* or in closely related species [10], [21], [23], [24], [41]. This includes two new male-specific compounds (6-D, 5-D), seven female-specific substances (8-P, 4-P, *x,x*-TD, *x,x*-PD, Br-M1, Br-M2, Br-M3) and eight CHs shared by both sexes (7-He, 5-He, 6-T, 4-T, 8-Te, 6-Te, 12-P, 24-Br). As we expected, no dienes were detected on the cuticle of virgin males. This is coherent with the sex-specificity of the enzymes involved in diene biosynthesis [42] but contradicts the recent data of Yew et al. [21].

### Validation of SPME

With the exception of cVA and long-chain CHs (>29C) which were not detected with SPME sampling of Drosophila cuticle, whole body solvent extraction and SPME sampling yielded only minor quantitative differences. Both direct GC-MS analysis of fly cuticular extracts and their indirect analysis via SPME detected cVA and long-chain CHs (>29C). This indicates that SPME can detect these compounds when they are present. We hypothesize that SPME detects the CHs present on the topmost layers of the fly cuticle, while solvent extracts compounds from more internal regions of the insect, which can differ from those present on the epicuticular surface [43], [44]. Long-chain CHs (31-Br, *n-*C31) and cVA may be located in deeper layers of the cuticle; this would explain why they are found only in the whole body solvent extract. Similar results and conclusions were found with the beetles *Megacyllene robiniae* and *M. caryae*. The comparison of cuticular hydrocarbon profiles obtained by whole body solvent extraction and by SPME sampling demonstrated that, in these two species, only the most abundant compound on the surface of the wax layer ((*Z*)-9-pentacosene and (*Z*)-9-nonacosene, respectively) is the female contact pheromone. In whole-body beetle extracts these compounds were mixed with other inactive hydrocarbons which were found only under the epicuticle [39], [40]. These results indicate that SPME-GC-MS provides an accurate description of the cuticular profile of the insects. Above all, it identifies those surface cuticular compounds that are truly available to other individuals, through gustatory or olfactory sensory neurons.

SPME has the important advantage of being non-destructive. This allowed us to repeat measurements of the same individual. This possibility of repeated measurement of the same individual has been used in ants to establish a correlation between CHs and reproductive [28], [29], [30] or social [31] status. SPME has also been used to demonstrate that CHs are involved in nestmate recognition in ants [32], and to investigate the relationships between a parasitic wasp and its host [45]. We used SPME to study the temporal dynamics of the hydrocarbon profile in Drosophila. Aging produced small but significant sex differences: in males, the amounts of most saturated and methyl-branched CHs decreased between 4 and 6 days; in females, most short-chain CHs decreased while both 5-P and 29-Br increased. Three short-chain compounds, 9-Te, 8-Te, and 7-Te, did not change in 6-day-old females whereas they significantly decreased in males of the same age. Variation in any of these compounds following mating is more likely to be due to aging than any putative pheromonal effect. However, we cannot exclude the possibility that these variations may be caused by rubbing the SPME fibre on the fly cuticle, through the partial removal of compounds, stress caused by manipulation, etc.

### Mechanical Exchange of Cuticular Compounds during Mating

Mating produced far more dramatic changes in CH profile. Most compounds showed a reciprocal variation between the sexes: the lighter compounds, which were predominant in males prior to mating (7-D, 6-D, 9-T and 7-T) decreased in males and increased in females, whereas the heavier compounds (9-H, 7-H, 7,11-PD, 7,11-HD and 7,11-ND), which were predominant in females prior to mating, varied in opposite direction. Several other hydrocarbons (5-D, 7,11-TD, 9,13-PD and *n-*C27) also showed an opposite variation, which was significant in only one sex. It seems most likely that this striking reciprocal variation is due to the mechanical transfer from one sex to the other during mating, as previously suggested for some male-specific compounds [24], [46]. The transfer of 7-T, and perhaps of other tricosenes, onto the female cuticle apparently modulates post-mating behaviour in females [24], [46], [47]; we hypothesize that post-mating variation in other male and female compounds may also have important behavioural consequences.

Such mechanical effects may also account for the apparent reversal of an age-related change seen in mated females: 5-P, *n-*C25, 27-Br and 29-Br decreased in mated females (but did not change in mated males) while they tended to increase with age in virgin females (Fig. 2C, D). The aging females apparently transferred some of their supply of these substances to their sexual partners.

### cVA and Sexual Interaction

Only cVA showed a parallel variation in both sexes: it was not detected in virgin flies of either sex and appeared in all mating males and females. Since its identification as a male-specific lipid in the Drosophila ejaculatory bulb over forty ytears ago [16], cVA has been described as an aggregation pheromone [48] and as a dual-purpose sex pheromone, inhibiting mating behaviour in males [19] but promoting mating behaviour in females [17]. Recently several studies have identified the molecular basis of cVA function and the circuitry underlying its behavioural effects [17], [18], [49], [50], [51].

Contrary to recent suggestions [21], [24], [52], [53], our data shown that cVA is *not* a cuticular component of virgin male flies. We suspect that this discrepancy may be due to the relatively invasive techniques used by previous studies. As discussed above, solvent extraction [52], [53] can release compounds from within the insect body, while DART and UV-LDI techniques [21], [24] and far from passive (see the movie in the supplemental data for Yew,2008 [24] and the Fig. 1G & H, in Yew, 2009 [21]) and both could elicit a leak of the ejaculatory bulb secretion onto the male cuticle.

Our study found no evidence that cVA is present on the cuticle of virgin males. We conclude that cVA cannot be considered as a pheromone that plays a role before copulation. It is emitted by the male during sexual interaction and mating and is transferred to the female during copulation. This may also be the case for CH503, which was recently detected on the anogenital area of male flies [21].

Ejima et al. [52] found that only females that copulated long enough to receive ejaculate (>14 min) had significant levels of cVA, even though they had significant amounts of passively acquired 7-tricosene. However, our data show that even if copulation is disrupted earlier, cVA can nevertheless be transferred from the male to the female. This is coherent with the findings of Scott and Richmond who detected an increase in cVA in females one min after copulation onset [54].

The roles of cVA as an aggregation pheromone and as a sex pheromone are context dependent. Strcitly speaking, cVA is not an aggregation pheromone: it attracts flies only when associated with food or food-derived odours [48]; on its own it has no behavioural effect. Its role as a sex pheromone is variable. It is stimulatory for females and inhibitory for males [17] and may require mature Drosophila CHs - not found on immature virgins - to synergise its anti-aphrodisiac effect [52]. Finally, in crowded conditions, cVA promotes male–male aggression, leading to the dispersion of male flies [55].

cVA is not found on isolated virgin males or females, but we hypothesize that a male courted by another male could emit some cVA (as found with DART or UV-LDI sampling) and this could inhibit male-male courtship. This could be related to the effect of social context on cVA production, which accounts for more than 50% of the variability in cVA levels [49].

### Mating Alters some Putative Pheromones

Several compounds varied in only one sex after mating, indicating that mechanical transfer is not the only effect that occurs during mating, and that other, physiological and/or pheromonal effects may occur. For example, 5-P sharply decreased in mating females, but increased in aging females; the related compounds 7-P and 9-P showed no such effect. Since both 7-P and 9-P have been implicated in the regulation of male copulatory behaviour [13], [14], the strong mating-dependent decrease in female 5-P may be due to the absorption of this substance by the male when he is licking the female genitalia during courtship.

More strikingly, 9-D, 6-Te and 26-Br decreased in mated males but were not affected in mated females, suggesting they were not simply passed from male to female. We hypothesize that this effect is due to a rapid change during courtship and mating, and that these compounds may be pheromones. Rapid quantitative variation in pheromonal levels has been postulated in *D. melanogaster* in a different social context [53]; females in several Drosophila species produce an anal droplet of volatile mating-stimulating material [56], [57], [58], and a similar phenomenon has been described in the closely related species *D. sechellia* [59].

Four of the compounds that were shown here to display striking unilateral post-mating variation (*n-*C25, 26-Br, 27-Br and 29-Br) have previously been identified as putative *ur*-pheromones, ancestral compounds shared by related species, which induce a non-species specific sexual excitation [15]. The fact that these substances show rapid, non-mechanical changes in their levels in individual flies following mating reinforces our hypothesis and provides further encouragement for our suggestion that the evolution of chemical communication in Drosophila involved both stimulatory (intraspecific) and inhibitory (inter- and intraspecific) aspects. Above all, our precise measures of individual variation in CH levels following mating reveal that the chemical conversation that takes place between male and female flies is far more complex than is generally accepted. They also indicate that the current tendency to focus on a single compound, while productive in the short term, will not provide a satisfactory understanding of the evolution and function of the chemical signature of Drosophila males and females.

## Materials and Methods

### Fly Husbandry

We used *Drosophila melanogaster* flies of the Dijon 2000 (Di2) wild-type strain [60]. Fly stocks were maintained on alcohol-free standard cornmeal medium mixed with killed yeast in 30 ml glass vials, at 24±0.5°C and 65±5% humidity on a 12∶12 dark∶light cycle. 1–2 hour old flies were sexed under light carbon dioxide anaesthesia 2–4 hours after lights on and were individually kept in fresh-food vials until 4 days old.

### Cuticular Hydrocarbon Extraction

CHs were first sampled using SPME from individual 4 day old male and female flies. Flies were then kept individually in fresh food vials for 2 days. At 6 days old, some of these flies were again sampled by SPME sampling after cold anaesthesia (1 min at −20°C) and were then individually extracted in hexane. The remaining 6 day old flies were placed in male-female pairs and allowed to mate. Immediately after mating began, the flies were cold anesthetized and separated using sharp tweezers; their CHs were then individually sampled using SPME. Experiments and controls were replicated 6 to 10 times.

#### Solid Phase Micro Extraction (SPME) of living flies

We first compared the effect of fibre polarity on the compounds collected by SPME, using an apolar fibre (carbowax/divinylbenzene) and a polar fibre (polydimethylsiloxane): both fibres collected all the compounds described here, and significant qualitative differences were observed for only a few compounds (two in male and three in female cuticular profiles) that were present in extremely small amounts (lower than 1%–See Tables 2 & 3). We therefore used a StableFlex fibre covered with carbowax/divinylbenzene (CW/DVB, 70 µm, Supelco, St Quentin-Fallavier, France). The fibre was first conditioned for 30 min at 230°C in the injection port of the gas chromatograph.

After the individual fly was cold anesthetized (1 min at −20°C), the full length of the fibre (±1 cm) was softly rubbed twice on the principal parts of its body (head, thorax, wings, abdomen, genitalia). The fibre was rotated slightly between each sample. Immediately afterwards, we checked that the fly was not injured, and then introduced the SPME fibre into the GC-MS injection port, using a manual Supelco SPME holder.

#### Whole body hexane extraction

Flies were individually plunged, at room temperature, for 5 min into vials containing 30 µl hexane with 100 ng n-hexacosane (*n*-C26) and 100 ng n-triacontane (*n*-C30) as internal standards (IS-1 and IS-2, respectively). These compounds were chosen because Di2 flies of both sexes lack these alkanes. After the fly was removed, the extracts were kept at −20°C until they were analysed using the same GC-MS conditions as for SPME.

#### SPME sampling of CHs in hexane extracts

The SPME fibre was immersed for 5 min at room temperature in a whole-body hexane extract. This extract was obtained by immersing four 6-day-old virgin flies for 5 min in 120 µl hexane with 400 ng of IS-1 and IS-2. The SPME fibre was introduced into the GC-MS injection port as described above, and a 1 µl aliquot of the hexane solution was then analysed by GC-MS.

### GC-MC Analysis

A QP2010 Shimadzu GC-MS apparatus in splitless mode, fitted with a VF-1ms fused silica capillary column (20 m×0.15 mm ID, 0.15 µm film thickness, Varian) was used. The column was held isothermally at 140°C, then programmed at a rate of 3°C/min to 300°C. Helium was used as the carrier gas at a linear velocity of 47 cm/sec. The injector port was set at 280°C. The mass spectrometer was operated at 70 eV and scanning was performed from 29 to 600 amu at 0.5 scans/sec. The injection split was opened 1 min after injection. The detected components were identified using their Kovats indices [61]; their fragmentation patterns and diagnostic ions were compared with both the NIST/EPA/NIH library and our own mass-spectrum library and compared with previously published Drosophila CHs.

### Statistical Procedures

All statistical tests were performed using XLSTAT 2007 [62]. We used the Wilcoxon signed-rank test for pairwise comparisons between the proportions of each compound (global analysis). Individual analysis was used to study individual cuticular compound variations as a function of aging or mating. For each compound we calculated the ratio of its relative abundance in each 6-day-old fly (virgin or mated) and in the same fly at 4 days (“*Post/Ante ratio*”). The null hypothesis was that CH proportions would not vary with age and that their *Post/Ante ratio* would be equal to 1. In both sexes, the ratios were grouped into two sets of data related to age and mating effects. The normality of each data set was measured using the Shapiro-Wilks W test and their coefficients of skewness were calculated [63]. We then calculated the confidence limits of the mean for each data set. Data were considered to be significantly different when at least 80% of individuals were outside these confidence limits.
